# SEDE-GPS: socio-economic data enrichment based on GPS information

**DOI:** 10.1186/s12859-018-2419-4

**Published:** 2018-11-30

**Authors:** Theodor Sperlea, Stefan Füser, Jens Boenigk, Dominik Heider

**Affiliations:** 1 Faculty of Mathematics and Computer Science, University of Marburg, Hans-Meerwein-Str. 6, Marburg (Lahn), D-35032 Germany; 2 Biodiversity Department, Center for Water and Environmental Research, University of Duisburg-Essen, Essen, D-45141 Germany

**Keywords:** GPS, Data enrichment, Database, Ecology, Microbial ecology

## Abstract

**Background:**

Microbes are essentail components of all ecosystems because they drive many biochemical processes and act as primary producers. In freshwater ecosystems, the biodiversity in and the composition of microbial communities can be used as indicators for environmental quality. Recently, some environmental features have been identified that influence microbial ecosystems. However, the impact of human action on lake microbiomes is not well understood. This is, in part, due to the fact that environmental data is, albeit theoretically accessible, not easily available.

**Results:**

In this work, we present SEDE-GPS, a tool that gathers data that are relevant to the environment of an user-provided GPS coordinate. To this end, it accesses a list of public and corporate databases and aggregates the information in a single file, which can be used for further analysis. To showcase the use of SEDE-GPS, we enriched a lake microbial ecology sequencing dataset with around 18,000 socio-economic, climate, and geographic features. The sources of SEDE-GPS are public databases such as Eurostat, the Climate Data Center, and OpenStreetMap, as well as corporate sources such as Twitter. Using machine learning and feature selection methods, we were able to identify features in the data provided by SEDE-GPS that can be used to predict lake microbiome alpha diversity.

**Conclusion:**

The results presented in this study show that SEDE-GPS is a handy and easy-to-use tool for comprehensive data enrichment for studies of ecology and other processes that are affected by environmental features. Furthermore, we present lists of environmental, socio-economic, and climate features that are predictive for microbial biodiversity in lake ecosystems. These lists indicate that human action has a major impact on lake microbiomes. SEDE-GPS and its source code is available for download at http://SEDE-GPS.heiderlab.de

**Electronic supplementary material:**

The online version of this article (10.1186/s12859-018-2419-4) contains supplementary material, which is available to authorized users.

## Background

The global positioning system (GPS), established in 1972 and made publicly available in 2000, allows for the exact identification of every spot on the surface of the earth [1]. Consequentially, when studying geographically localized objects or processes such as ecosystems, their location can easily be specified using GPS coordinates.

Many natural processes are strongly influenced by characteristics of their surroundings, i.e., it is known that chemical composition, size of different habitats, and socio-economic features such as human population size, can influence the (microbial) biodiversity in ecosystems [2–5]. Therefore, having access to environmental characteristics and including them in analyses is crucial when trying to understand natural processes.

In the current study, we describe the novel tool SEDE-GPS (**S**ocio-**e**conomic **d**ata **e**nrichment based on **GPS** information), which can be used to enrich data sets with data from public and publicly available corporate databases based on user-specified GPS information. The current version of SEDE-GPS accesses Open Street Map (OSM), the Climate Data Center (CDC), Eurostat, and Twitter. SEDE-GPS has an easy-to-use graphical user interface and enables researchers to enrich their data with environmental and socio-economic information based on GPS information. This may lead to new insights into the influence of environmental and socio-economic features on a wide range of processes.

As an exemplary use-case of SEDE-GPS, we use it in order to identify features that have an impact on microbial biodiversity. To this end, we calculate different alpha diversity metrics from a sequencing dataset sampled from a set of alpine lakes in Austria. We then use feature selection and machine learning methods to determine features from the output of SEDE-GPS that can be used to predict these alpha diversity metrics. Our results show that both microbial Eukaryotes and Prokaryotes are impacted by different environmental features. Nevertheless, for both domains, the area and number of city structures (or lack thereof) and other human-related features carry high predictive power.

## Implementation

SEDE-GPS can be used via both a graphical user interface (GUI) and a command line interface. As main input, SEDE-GPS takes a list of at least one GPS coordinate. Additionally, SEDE-GPS needs a set of parameters specifying which databases will be queried and restrictions on the subfields to be downloaded. In the GUI, these parameters can be selected via mouse-click, however, in the command line version, these parameters need to be specified in a config file. The output of the different modules implemented in SEDE-GPS is temporarily saved and removed after being merged to a final output file in the csv format. This is due to the fact that the output of SEDE-GPS can be too large for regular-sized memory.

**Fig. 1:**
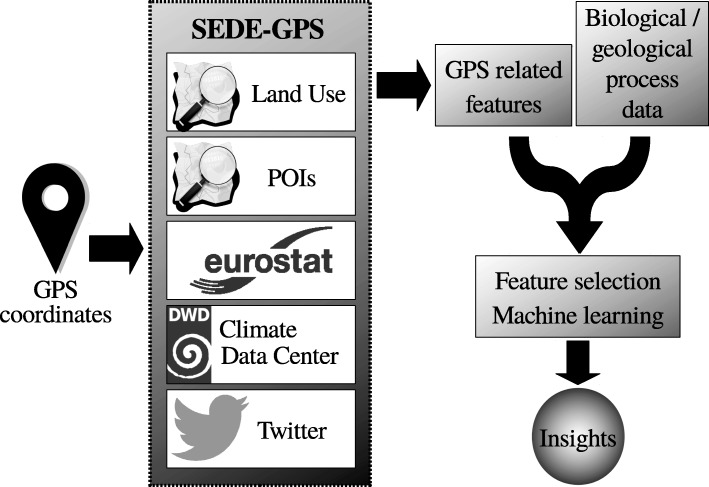
Sample workflow for the use of SEDE-GPS. Based on user-defined GPS positions, SEDE-GPS queries a set of modules and returns all relevant data. This data can then be used in analyses of any geo-located process. Due to the huge amount of features present in the dataset after data enrichment with SEDE-GPS, we recommend including a feature selection step before using the gathered data for model construction, e.g., based on machine learning. Data sources are represented by their respective logos which were taken from Wikimedia (https://commons.wikimedia.org/wiki/Main_Page)

In the following, we will discuss the sources for data enrichment currently used by SEDE-GPS (Fig. 1).

***OSM: Land use statistics***

Open Street Map (OSM) is a community-generated, worldwide map. It is used by SEDE-GPS to gather information on land-use of the area that surrounds a given GPS position [6]. An area with an user-defined perimeter is extracted from relevant map tiles of the OSM database. As OSM maps are represented in Mercator projection, SEDE-GPS compensates for latitudinal distortion. From this map excerpt, the relative amount of pixels covered by different map legend objects are calculated by thresholding for their respective colors. This will calculate the fraction of area around the user-provided GPS position that is covered by, e.g., forests, city structures, or bodies of water.

***OSM: POIs***

In addition to the map itself, OSM also hosts a database that contains the locations of specific points of interests (POIs), such as special buildings or touristically relevant objects [6]. This module queries the OSM API and counts the number of the different POIs in a perimeter of user-defined size around the GPS coordinates. As the OSM API reacts to queries slowly, this module is the largest contributor to the runtime of SEDE-GPS. Therefore, for larger analyses, it is advisable to manually download the so-called planetfile from OSM and to use it as an additional input for SEDE-GPS.

***Eurostat: detailed regional statistics***

The Eurostat database contains highly detailed governmentally collected data from the EU and EFTA member states [7]. Its regional database provides statistics on economic and social composition of centrally defined NUTS (*Nomenclature des unités territoriales statistiques*) regions. This module first determines the NUTS region that corresponds to the user-specified GPS position by querying the Google Maps database for the GPS positions’ postal code. With around 17,500 features, this module’s output represents 99.4% of all features gathered by SEDE-GPS.

***CDC: European climate data***

Via the CDC, a ftp server mainained by the Deutscher Wetterdienst (DWD), it is possible to publicly and freely access European climate data that dates back to 2010 [8]. The data has an interpolated spatial resolution of 5 km and a chronological resolution of a day or a month. This module requires a date as additional input and calculates average values of, e.g., temperature or windiness for the specified day, month, and/or year.

***Twitter***

The short messages sent out by users of Twitter (so-called tweets) can be location-tagged, and their number can be used to estimate tourist interest in a POI. The Twitter module of SEDE-GPS collects and counts tweets sent from a user-specified perimeter around the GPS coordinates. Twitter limits the access to its data so that SEDE-GPS can access all tweets that were sent in the last 7 days, but can only send 75 queries per 15 min. For a large number of GPS coordinates, this module will, therefore, require a long runtime.

## Methods

### Calculation of alpha diversity indices

The sequence data analyzed in the current study was taken from [9, 10] (Additional file 1). It stems from a set of alpine Austrian lakes, which were sampled in order to study the change of lake microbial ecosystems of three different lakes over time [9] and the difference in microbiome composition over many lakes [10]. 16s and 18s SSU rRNA sequences were sequenced using a 454 deep-sequencing amplicon approach [9, 10]. In the current study, only samples that were taken in August 2006 and contain more than 1000 sequences were analyzed. 16s and 18s rRNA sequences were analyzed separately.

In order to estimate biodiversity within the samples, we calculated four different alpha diversity indices, namely Shannon’s Entropy *H*^′^, Simpson diversity *D*, Simpson evenness *E*, and the Chao1 Estimator *C*, at the maximum possible sequencing depth with QIIME [11]. These indices describe the mean species richness or diversity at the local level [12] and are described by the following equations:

1 $$\begin{array}{@{}rcl@{}} H' = - \sum\limits_{i=1}^{R} p_{i} \ln p_{i} \,\,\,\,\,\, with ~~~~~ p_{i} = \frac{n_{i}}{N} \end{array} $$

2 $$\begin{array}{@{}rcl@{}} D = 1 - \frac{{\sum\nolimits}_{i=1}^{R} n_{i} (n_{i} - 1)}{n(n-1)} \end{array} $$

3 $$\begin{array}{@{}rcl@{}} E = -\frac{1/\lambda}{R} \,\,\,\,\,\, with ~~~~~ \lambda = \sum\limits_{i=1}^{R} \left(\frac{n_{i}}{N}\right)^{2} \end{array} $$

4 $$\begin{array}{@{}rcl@{}} C = R + \frac{S_{1}(S_{1}-1}{2(S_{2}+1)} \end{array} $$

where *R* is the number of species, *n*_*i*_ the number of individuals in species i, *N* the total number of individuals, *S*_1_ the number of singletons (i.e., the number of species with only one individuum), and *S*_2_ the number of doubletons (i.e., the number of species with exactly two individuals).

### Feature selection and feature evaluation

Before using the output of SEDE-GPS for machine learning, we employed a feature selection step. To this end, features containing missing values and with low variance (e.g., with more than 25% zeroes) were discarded. Next, we used the R package EFS (Ensemble Feature Selection) in order to rank the remaining features according to their importance. EFS is an ensemble learning feature selection method, that corrects for biases of the single methods when weighting features [13, 14]. Although EFS has been developed for feature selection in classification studies, we used an adapted version of EFS, which can be used for regression studies.

Stability of the features gathered over multiple runs of EFS were assessed by calculating the mean pairwise distance between the feature lists. To this end, we calculated Kendall’s *τ* and the Jaccard distance using the R packages *kendall* and *philentropy* [15, 16]. For two ranked lists of observations *x* and *y* of length *n*, Kendall’s *τ* is defined as

5 $$\begin{array}{@{}rcl@{}} \tau(x,y) = \frac{c-d}{n(n-1)/2} \end{array} $$

with *c* being the number of pairs of concordant observations (*x*_*i*_,*y*_*i*_) and (*x*_*j*_,*y*_*j*_) with *x*_*i*_<*x*_*j*_ and *y*_*i*_<*y*_*j*_, *d* the number of discordant observations with

6 $$\begin{array}{@{}rcl@{}} \left(x_{i} > x_{j}\right) \& \left(y_{i} < y_{j}\right) \parallel \left(x_{i} < x_{j}\right) \& \left(y_{i} > y_{j}\right), \end{array} $$

*i* and *j* indices in the lists *x* and *y*, respectively.

The Jaccard distance *d*_*J*_ for two lists *x* and *y* is defined as

7 $$\begin{array}{@{}rcl@{}} d_{J}(x,y) = \frac{|x \cup y| - |x \cap y|}{|x \cup y|}. \end{array} $$

Therefore, for two feature lists with a maximum distance, the Jaccard distance would assume a value of 1 and Kendal’s *τ* a value of −1. These values were calculated from feature lists that contain the 50 features that were ranked most important by EFS.

Sets of correlating features were determined using Spearman correlation at a correlation coefficient cutoff of larger than 0.7.

### Machine learning

We trained and evaluated eleven different machine learning models (as implemented in the R package *caret* [17]) using a leave-one-out cross-validation (LOOCV) scheme. These models included generalized linear models (*glmnet*), bayesian lasso (*blasso*), support vector machines (*svmLinear* and *svmRadial*), k-nearest neighbors (*knn*), Regression Trees (CART: *rpart*, bagged CART: *treebag*), Random Forests (*rf*), and stochastic and extreme gradient boosting (*gbm* and *xgbTree*). Models were evaluated by comparing the predicted values for all iterations to the real alpha diversity values, resulting in *R*^2^ values. Confidence intervals for the models’ performance were calculated from the distribution of *R*^2^ values that were gathered from 1000x bootstrapped pairs of predicted and observed target variables. Their distributions were visualized using boxplots.

The machine learning models were tested for overfitting using a permutation test. To this end, the target variable was permutated and after feature selection with EFS, mache learning models were trained using the same approach as described above. *R*^2^ values were calculated and collected for 1000 repetitions of this procedure. Finally, the number of times *t* the resulting *R*^2^ value is larger than or equal to the *R*^2^ value received with an unpermutated target variable was counted. Significance in terms of a p value was calculated by *p*=*t*/1000.

## Results

***Data enrichment using SEDE-GPS***

 SEDE-GPS is structured modularily, with every module querying a certain database or API and, if necessary, data pre- and postprocessing steps (Table 1). The modules that query the Open Streetmap (OSM) databases, e.g., have to account for the fact that their maps are in a Pseudo-Mercator projection or calculate a bounding box for counting of POIs. Some of the APIs queried by SEDE-GPS limit the number of queries that are handled in a certain amount of time (Twitter) or answer intentionally slowly (OSM). Similarly, the number of features provided by the different modules varies greatly, with Eurostat contributing by far the most the highest number of features, respectively (Table 1).

**Table 1:** Modules and their subfields currently available in SEDE-GPS

Module	Subfields	Additional Input	Data Processing	No. of features	Runtime (ms)
OSM Land Use	-	Radius	Pixel decompression	20	24823 ±2421
OSM POIs	Craft	Radius	Bounding boxes	7	3229 ±342
	Leisure	Radius	Bounding boxes	15	7202 ±622
	Powerplants	Radius	Bounding boxes	11	5053 ±503
	Special buildings	Radius	Bounding boxes	13	6881 ±453
	Tourism	Radius	Bounding boxes	8	3096 ±382
	Transport	Radius	Bounding boxes	13	6951 ±496
	Urban	Radius	Bounding boxes	6	2402 ±401
CDC	Average of the day	Date		4	<1
	Average of the month	Date		4	2 ±0
	Average of the year	Date		4	211 ±0
Eurostat	Agriculture			721	711 ±80
	Business Demography			778	1467 ±83
	Crime Statistics			4	16 ±4
	Demography			15077	2611 ±79
	Economic Accounts			67	431 ±41
	Education Stat.			30	31 ±5
	Labour Market Stat.			99	172 ±17
	Science & Technology			644	3718 ±400
	Tourism Stat.			44	163 ±11
	Transport			59	13383 ±224
Twitter	-	Radius		1	1014 ±316
Total				17629	83567

Runtime means and standard deviation were calculated from ten measurements

In order to showcase the use of SEDE-GPS, we planned to identify features that are predictive for the microbial biodiversity in a set of 39 alpine Austrian lakes. From these lakes, water samples were taken from which both 16s and 18s rRNA were sequenced and the geo-location of the sampling was recorded using GPS [9, 10]. These GPS coordinates were used as an input for SEDE-GPS, with all modules enabled, using radii of 1, 2, and 5 km and the date of sampling as additional input for modules for which this is necessary. This resulted in around 17,900 features.

The resulting dataset was observed to be highly sparse, with especially the output of the Eurostat and Twitter module showing a high degree of sparsity. Furthermore, a very small amount of features contained missing values, which we attributed to either errors in the databases or in the communication with the API. Therefore, features were discarded that contained any missing values or zeroes for more than a third of the instances. This procedure reduced the number of features per lake to around 1,200.

***Calculation of biodiversity metrics***

The 16s and 18s rRNA sequencing datasets were processed separately using a QIIME pipeline [11]. Samples that contained less than 1000 sequences were discarded, which lead to differing numbers of lakes for which Eukaryotic and Prokaryotic biodiversity data were available. As biodiversity indicators, four different Alpha diversity metrics (Shannon’s entropy, Simpson diversity, Simpson evenness, and the Chao1 estimator) were calculated after rarefaction (“Methods” section). We used multiple different metrics as they each measure biodiversity in specific ways and therefore emphasize different species distribution characteristics [18–20]. As the alpha diversity metrics were calculated for 16s and 18s rRNA separately, this resulted in maximally eight different biodiversity indicators for each lakes.

***Identification of important features using EFS***

In order to find features in the output of SEDE-GPS that are predictive for lake microbial biodiversity, we used the R package EFS (Ensemble Feature Selection) and the eight alpha diversity metrics as target variable in separate analyses [13, 14]. EFS is an ensemble feature selection method that assigns weights to the features in an unbiased manner according to their predictiveness for the target value.

**Fig. 2:**
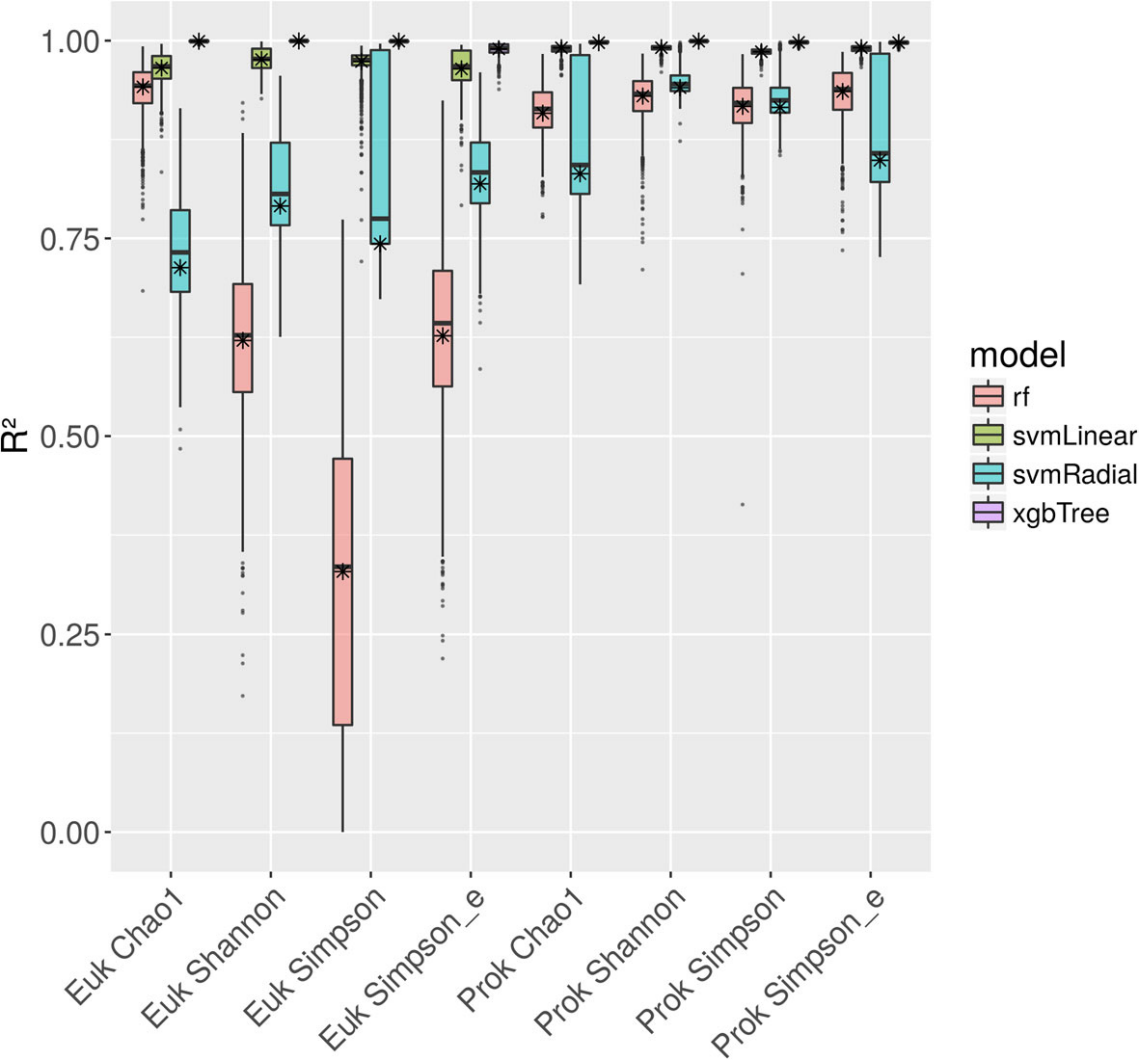
Performance of machine learning models predicting microbial lake alpha diversity based on the output of SEDE-GPS. Stars represent the performance of models trained on the respecitve dataset, box plots represent confidence intervals of *R*^2^ values gathered from the respective model. Models were trained on the output of SEDE-GPS after feature selection and evaluated using LOOCV (“Methods” section). Only results for the four best-performing models are shown; for the others, see Table 2

Using the average weight of the features as cutoff, features below this cutoff were discarded. To verify that the selected features are both descriptive and were not selected due to overfitting, eleven different machine learning models were trained to predict the eight alpha diversity values from the EFS-selected SEDE-GPS features. The models showed profoundly differences in performance (Table 2) with *xgbTree* showing near perfect performance for all target variables (Fig. 2). In order to confirm that the performance of the models is not due to overfitting, we performed a permutation test for the four best-performing machine learning models. For all target variables and machine learning models, this resulted in a p-value of less than 0.001.

**Table 2:** Performance (*R*^2^ values) of machine learning models trained to predict alpha diversity from SEDE-GPS output

Dataset	*glmnet*	*blasso*	*svmRadial*	*svmLinear*	*knn*	*rpart*	*treebag*	*rf*	*gbm*	*xgbTree*
Euk Chao1	0.292	0.003	0.713	0.980	0.0415	0.214	0.631	0.518	0.496	0.999
Euk Shannon	0.228	0.0167	0.791	0.993	0.000	0.180	0.635	0.582	0.680	1.000
Euk Simpson_e	0.277	0.0146	0.556	0.976	0.107	0.238	0.671	0.559	0.546	0.980
Euk Simpson	0.150	0.001	0.742	0.906	0.014	0.090	0.545	0.346	0.432	0.995
Prok Chao1	0.768	0.461	0.832	0.991	0.0695	0.420	0.635	0.915	0.955	0.979
Prok Shannon	0.527	0.011	0.940	0.991	0.172	0.538	0.626	0.930	0.993	0.999
Prok Simpson_e	0.345	0.128	0.849	0.991	0.035	0.304	0.622	0.937	0.840	0.999
Prok Simpson	0.459	0.008	0.915	0.986	0.168	0.453	0.627	0.904	0.880	0.991

Taken together, these results show that the features selected by EFS were not selected due to overfitting but are helpful for predicting alpha diversity metrics for prokaryotes and microbial eukaryotes in lakes.

***Stability and importance of features***

**Fig. 3:**
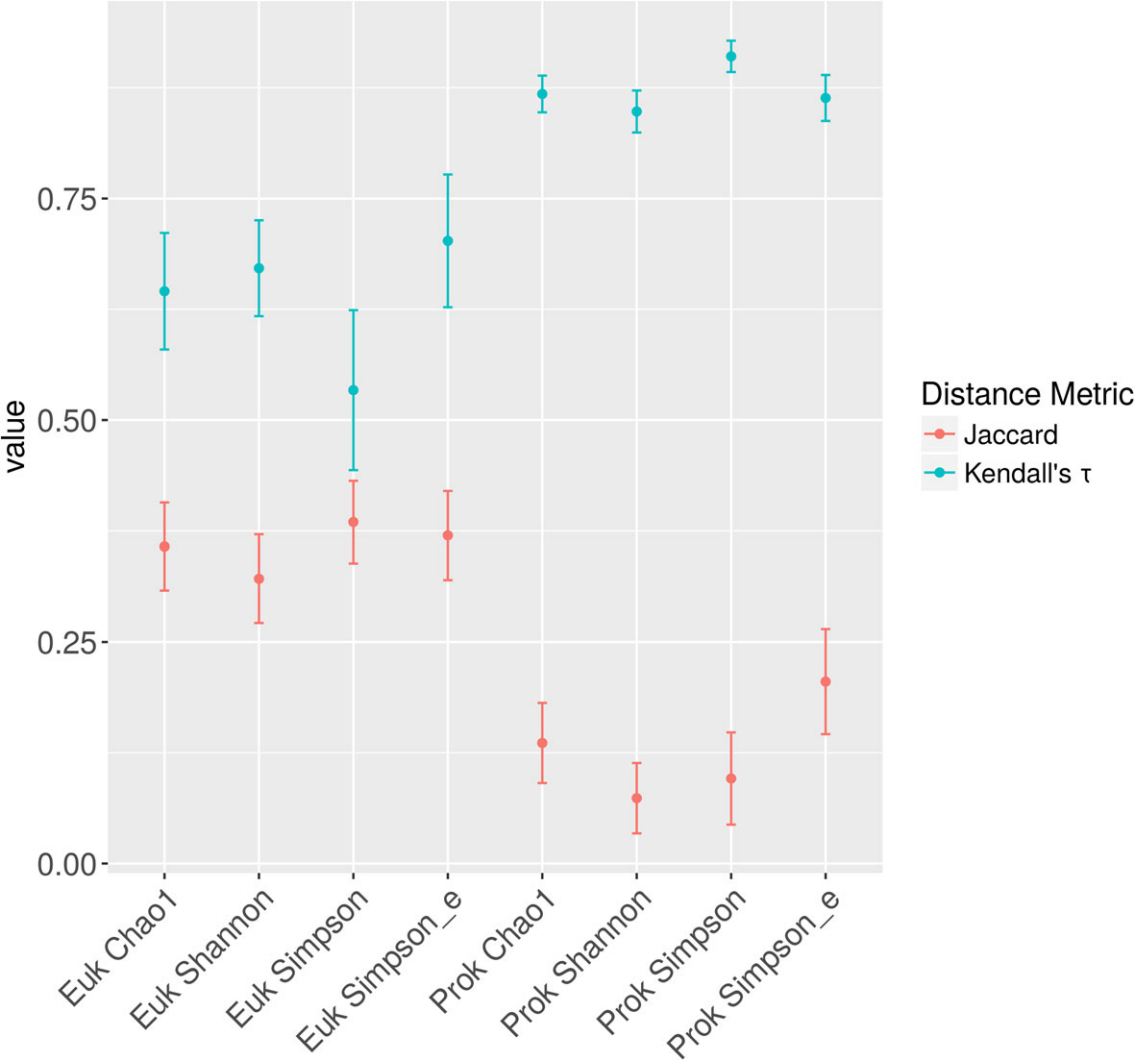
Stability of feature lists over LOOCV iterations. Jaccard distances and Kendall’s *τ* were calculated for pairs of feature lists for the 50 most important features of each dataset. Dots and error bars represent average values and standard deviations of values, respectively. At maximum distance, the Jaccard distance and Kendall’s *τ* would assume a value of 1 and −1, respectively. Both feature lists are rather stable, however, the feature lists of the Prokaryote datasets are more stable than their Eukaryote counterparts

Due to the fact that leave-one-out cross validation (LOOCV) was used to train and validate the machine learning models, multiple weighted feature lists were calculated for every target variable. Overfitting of EFS would have resulted in drastically different feature weights in the LOOCV iterations. In order to show that EFS did not overfit in the analyses presented here, we assess the stability of the features selected in the LOOCV iterations using both Kendall’s *τ* and Jaccard distance as feature list distance measures. These results show that the features selected by EFS show a high degree of stability and that the feature selection is not the result of overfitting (Fig. 3).

**Fig. 4:**
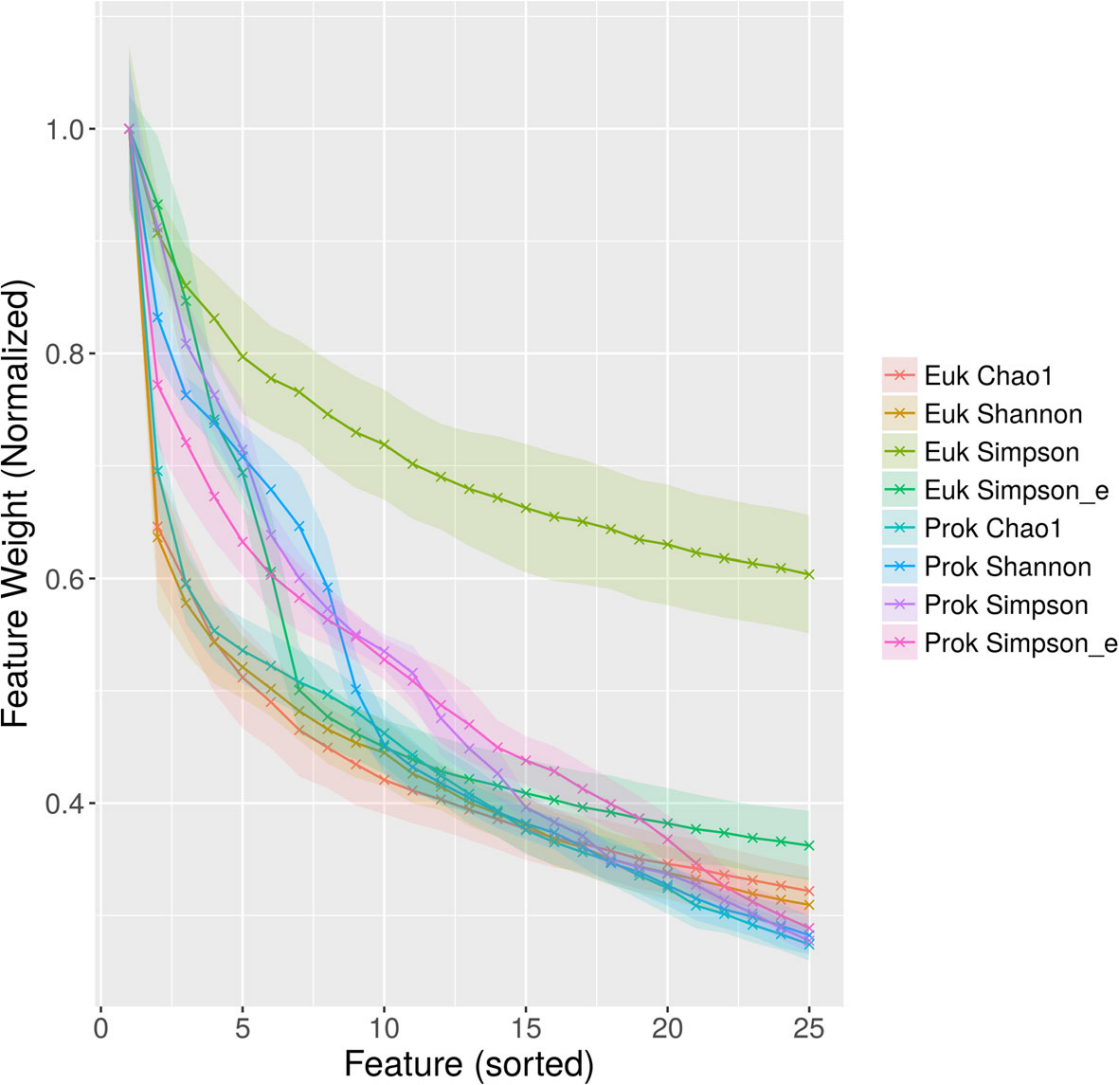
Decline of average importance of features over the 25 highest ranked features. Feature weights were calculated using EFS and averaged over the LOOCV iterations. Ribbons indicate standard deviation. Average importance values were normalized so that the first feature has an average weight of 1. For all datasets except Euk Simpson, after the twelfth highest weighted features, feature weights are below 0.5

When manually examining selected features, it is important to keep in mind that the first step of feature selection in EFS is correlation based. This means that from sets of features that correlate, only the most descriptive feature is kept in the feature set. Therefore, for datasets processed with EFS, each feature label must be viewed as stand-in for a set of correlating features. Table 3 shows the five most important features for predicting the different alpha diversity metrics, with each feature name being replaced by higher order descriptions of the respective set of correlating features (for the simple feature names, see Additional file 2: Table S1). This examination was limited to five features per target variable because both the average feature weight and the stability of the feature position decrease quickly with increasing rank of the feature (Fig. 4, Additional file 3: Figure S1).

**Table 3:** Features with the highest weights for prediction of different alpha diversity metrics for Prokaryotes and Eukaryotes in Austrian lakes

Prokaryotes			
Chao1	Shannon Entropy	Simpson Diversity	Simpson Evenness
Industrial Area, Villages, Street (2-5 km)	Forests (5km)	Forests (5km)	Forests (5km)
Forests (5km)	Main street (small), married people	Forests	Main street (small), married people
Climate, Demography, City Structures	Forests (2km)	Buildings, Highways, Water, Parking, Parks	Forests (1km)
Climate, Demography, City Structures	Climate, Demography, City Structures	Forests (1km)	Buildings, Highways, Water, Parking, Parks
Main street (small), married people	Green space, small villages, Industrial area	Mining, main streets	Mining, main streets
Eukaryotes			
Chao1	Shannon Entropy	Simpson Diversity	Simpson Evenness
Forests	Main streets	Main streets	Economy (parking, GDP, Agrarian structures), Population
Family Demography	Beach & Water	Beach & Water	Economy (parking, GDP, Agrarian structures), Population
Climate, Demography, City Structures	Picnic Site (5km)	Economy (parking, GDP, Agrarian structures), Population	Beach & Water
Altitude, Climate, Demography, City Structures	Highway Pull-ins	Towns	Towns
Climate, Demography, City Structures	Urban regions, Av. Temperature, Parks	Urban regions, Av. Temp., Parks	Highway Pull-ins

For features in bold, a linear regression shows a positive relationship with the respective target variable

The resulting feature lists for Prokaryotes and microbial Eukaryotes show major differences, while using different alpha diversity metrics result, especially for Prokaryotes, in similar feature lists (Table 3).

## Discussion

### SEDE-GPS

In this paper, we present SEDE-GPS, which can be used to drastically increase the number of features for datasets that contain GPS-located samples. Accessing four different data sources via five modules, it provides around 18,000 numerical features that contain socio-economic, geographic, and climate information (Table 1).

Currently, due to the choice of databases SEDE-GPS queries, this tool has a number of limitations. Both the CDC and Eurostat modules return only data for GPS coordinates in Europe, while the OSM modules and Twitter module will work for any GPS coordinate. Similarly, the databases queried by SEDE-GPS do not contain meaningful data for most marine GPS coordinates. In the future, we seek to overcome these limitations by including more data sources and thus extending SEDE-GPS both to new regions and to new data types and formats.

Similarly, the specific limitations and pecularities of the databases currently used by SEDE-GPS are important for the interpretation of their data. OSM contains user-generated and user-curated information which might be of inconsistent albeit high quality or level of detail [6]. Eurostat, as a governmentally curated database, on the other hand, exhibits a level of detail which is generally lower that that of OSM as it can only be queried for defined NUTS regions [7]. As these regions are of widely differing sizes one might want to normalize data gathered from Eurostat to the area of the respective NUTS region. We decided not to implement this normalization step in SEDE-GPS as postprocessing steps not accessible to the user generally might introduce unwanted artifacts. The information gathered from Twitter comes with multiple caveats: For one, only very few processes will be directly influenced by the number of messages sent via Twitter and this number will thus, in most cases, function as a proxy for other information. Additionally, the number of tweets will show a certain amount of variance over time, with the amount of variance being possibly also location-dependent.

Because of a rate limitation in API queries, both the OSM modules and the Twitter module are the biggest contributors to SEDE-GPS’s runtime, especially for datasets with many GPS coordinates. It would be possible to speed up the OSM modules by reading the data from a so-called planetfile (an image of the OSM databases) instead of using API queries. This is, currently, not implemented in SEDE-GPS, as the planetfile is very large and a speed improvement would, therefore, only exist for very large GPS datasets.

Central to the design of SEDE-GPS is the fact that it does not perform any field-specific data postprocessing. Therefore, the output of SEDE-GPS can be used for studies in a wide variety of scientific fields. Nevertheless, for some applications, postprocessing steps might be advisable.

### Microbial ecology

In this study, we showcase the use of SEDE-GPS for microbial ecology. From the output of SEDE-GPS and using machine learning methods, we were able to identify features that can be used as predictors of both Eukaryote and Prokaryote alpha diversity in a set of alpine lakes.

Implicitly, in this study, we assumed that environmental features have a bigger impact on microbial biodiversity than historical contingencies and recent events. We acknowledge that this notion, succinctly formulated as “everything is everywhere, but the environment selects”, is highly debated [21–24]. Furthermore, we do not take into account that the composition of microbial communities can be majorly influenced by recent events or the microenvironment of the sampling position [25, 26]. These assumptions are neccesary because the dataset analyzed here does not contain multiple samples that were collected on different time points for each of the lakes. However, we are not aware of such an ecological microbial sequencing dataset with a quality, geographic extensiveness, and also uniformity of sample preparation comparable to the one we analyzed here.

The features we identified as most predictive for microbial biodiversity differed greatly between Eukaryotes and Prokaryotes, supporting the notion that microorganisms from these domains have different ecological roles [21, 24, 27, 28]. In contrast to this, the most predictive features for the different alpha diversity indices calculated from Prokaryotic sequences show a high degree of similarity. This indicates that the alpha diversity metrics used in this study essentially capture the same central distribution characteristics of the composition, at least for this domain of life.

Recently, many studies identified environmental and geographic features such as temperature, pH, climate, ion and nutrient concentration, and elevation-related environmental parameters as major drivers of the composition of lake microbiomes [4, 10, 21, 29–31]. Some of these features were also identified as highly impactful in our analysis (Table 3), albeit somewhat hidden under feature labels such as “Climate, Demography, City Structures” for temperature or “Economy (parking, GDP, Agrarian structures), Population” for nutrient concentration. While this clearly is a consequence of the field-agnostic nature of the data provided by SEDE-GPS, it might also point to possible sources for impact on biodiversity.

Therefore, our results also suggest that human action has an direct or indirect impact on lake micrbiome composition. Although an impact of urbanization on biodiversity is well known for other areas of ecology [32–35], this is the first time, to our knowledge, that it has been described for microorganisms. Surprisingly, our results suggest that urbanization has a positive effect on Prokaryote biodiversity, as, e.g., the area of the environment covered by streets correlates positively with all biodiversity indices used in this study (Table 3). The negative impact of forest area might therefore stem from the fact that areas covered with forests cannot also be urban regions. Importantly, one should not fall into the trap of assuming that a higher biodiversity necessarily signifies a well-functioning ecosystem [20] and take the results presented here to mean that more streets would improve lake ecosystems. Nevertheless, these results indicate that the processes that govern microbial ecology are very different from those that regard the ecology of larger organisms [9, 21, 28].

Further analyses will be needed to solidify the results of this study. In part, this is due to the fact that the samples and lakes included in this analysis are limited in number and are geographically close to each other [22, 24, 25, 36]. Therefore, for a more thorough analysis, larger datasets from more variable sites would be neccessary, as currently only available from large-scale environmental sequencing efforts such as the Earth Microbiome Project [37] or the 1000 Springs Project [28, 38]. Nevertheless, on the basis of the results presented here, experiments can be designed in order to illuminate the mechanistic and causal relationships between environmental features and microbial biodiversity.

## Conclusion

This study shows how to use SEDE-GPS in order to enhance datasets that contain scarce amounts information on the environment of geo-located, observed processes. Analysing the output of SEDE-GPS leads to the identification of environmental, socio-economical, and climate features that influence the studied process. These results can then act as basis for further hypothesis-driven research projects. SEDE-GPS is available at http://www.SEDE-GPS.heiderlab.de.

## Availability and Requirements

**Project name:** SEDE-GPS **Project home page:**http://www.SEDE-GPS.heiderlab.de**Operating system(s):** Platform independent **Programming language:** Java **License:** GNU GPLv3 **Any restrictions to use by non-academics:** None

## Additional files

Additional file 1 This table contains names, positions, and references for the samples contained in the sequence dataset and whether Prokaryotes and/or Eukaryotes were analyzed from the sample in this study. (CSV 3 kb)

Additional file 2 This table contains the feature names of the ten most important features in respect to the different alpha diversity metrics for Prokaryotes and Eukaryotes. Here, feature names were not replaced as described in “Methods” section. (CSV 2 kb)

Additional file 3 This figure shows the relative frequency of the most frequent feature at a given position for all target variables. Frequencies were calculated from the feature lists sorted by the weights determined by EFS in the LOOCV iterations. This shows that feature lists get more random with increasing rank of the feature on a sorted feature list. (TIF 844 kb)

## Abbreviations

CDC: Climate data center
GPS: Global positioning system
Lat: Latitude
Lon: Longitude
LOOCV: Leave-one-out cross validation
OSM: Open street map
POI: Point of interest

## References

[1] Parkinson B, Spilker J, Elkaim G. Global Positioning System (GPS) In: Mark H, editor. Encyclopedia of Space Science and Technology: 2003. 10.1002/0471263869.sst069.

[2] Vitousek PM, Mooney HA, Lubchenco J, Melillo JM. Human domination of earth’s ecosystems. Science. 1997; 277(5325):494–9. 10.1126/science.277.5325.494. http://arxiv.org/abs/http://science.sciencemag.org/content/277/5325/494.full.pdf.

[3] Ruan Q, Dutta D, Schwalbach MS, Steele JA, Fuhrman JA, Sun F. Local similarity analysis reveals unique associations among marine bacterioplankton species and environmental factors. Bioinformatics. 2006; 22(20):2532–8. 10.1093/bioinformatics/btl417. 16882654

[4] Hering D, Borja A, Carstensen J, Carvalho L, Elliott M, Feld CK, Heiskanen A-S, Johnson RK, Moe J, Pont D. The european water framework directive at the age of 10: A critical review of the achievements with recommendations for the future. Sci Total Environ. 2010; 408(19):4007–19. 10.1016/j.scitotenv.2010.05.031. 20557924

[5] Grossmann L, Beisser D, Bock C, Chatzinotas A, Jensen M, Preisfeld A, Psenner R, Rahmann S, Wodniok S, Boenigk J. Trade-off between taxon diversity and functional diversity in european lake ecosystems. Mol Ecol. 2016; 25(23):5876–88. 10.1111/mec.13878. 27747959

[6] OpenStreetMap contributors. Planet dump retrieved from https://planet.osm.org. 2017. https://www.openstreetmap.org. Accessed 15 Jan 2018.

[7] Eurostat. Eurostat database. 2017. http://ec.europa.eu/eurostat/data/database. Accessed: 21 Dec 2017.

[8] Deutscher Wetterdienst. Climate Data Center hosted by Deutscher Wetterdienst. 2017. ftp://ftp-cdc.dwd.de/pub/CDC/. Accessed: 21 Dec 2017.

[9] Nolte V, Pandey RV, Jost S, Medinger R, Ottenwälder B, Boenigk J, Schlötterer C. Contrasting seasonal niche separation between rare and abundant taxa conceals the extent of protist diversity. Mol Ecol. 2010; 19(14):2908–15. 10.1111/j.1365-294x.2010.04669.x. PMC2916215 20609083

[10] Grossmann L, Jensen M, Pandey RV, Jost S, Bass D, Psenner R, Boenigk J. Molecular investigation of protistan diversity along an elevation transect of alpine lakes. Aquat Microb Ecol. 2016; 78(1):25–37. 10.3354/ame01798.

[11] Caporaso JG, Kuczynski J, Stombaugh J, Bittinger K, Bushman FD, Costello EK, Fierer N, Peña AG, Goodrich JK, Gordon JI, Huttley GA, Kelley ST, Knights D, Koenig JE, Ley RE, Lozupone CA, McDonald D, Muegge BD, Pirrung M, Reeder J, Sevinsky JR, Turnbaugh PJ, Walters WA, Widmann J, Yatsunenko T, Zaneveld J, Knight R. QIIME allows analysis of high-throughput community sequencing data. Nat Methods. 2010; 7(5):335–6. 10.1038/nmeth.f.303. PMC3156573 20383131

[12] Whittaker RH. Vegetation of the siskiyou mountains, oregon and california. Ecol Monogr. 1960; 30(3):279–338. 10.2307/1943563.

[13] Neumann U, Riemenschneider M, Sowa J-P, Baars T, Kälsch J, Canbay A, Heider D. Compensation of feature selection biases accompanied with improved predictive performance for binary classification by using a novel ensemble feature selection approach. BioData Min. 2016; 9(1). 10.1186/s13040-016-0114-4. PMC5116216 27891179

[14] Neumann U, Genze N, Heider D. EFS: an ensemble feature selection tool implemented as R-package and web-application. BioData Min. 2017; 10(1). 10.1186/s13040-017-0142-8. PMC5488355 28674556

[15] McLeod AI. Kendall: Kendall Rank Correlation and Mann-Kendall Trend Test. 2011. https://CRAN.R-project.org/package=Kendall. Accessed 15 Jan 2018. R package version 2.2.

[16] Drost H-G. Philentropy: Similarity and Distance Quantification Between Probability Functions. 2017. https://CRAN.R-project.org/package=philentropy. Accessed 15 Jan 2018. R package version 0.0.3.

[17] Kuhn M. Building predictive models in R Using the caret package. J Stat Softw. 2008; 28(5). 10.18637/jss.v028.i05.

[18] Hill TCJ, Walsh KA, Harris JA, Moffett BF. Using ecological diversity measures with bacterial communities. FEMS Microbiology Ecology. 2003; 43(1):1–11. 10.1111/j.1574-6941.2003.tb01040.x. 19719691

[19] Morris EK, Caruso T, Buscot F, Fischer M, Hancock C, Maier TS, Meiners T, Müller C, Obermaier E, Prati D, Socher SA, Sonnemann I, Wäschke N, Wubet T, Wurst S, Rillig MC. Choosing and using diversity indices: insights for ecological applications from the german biodiversity exploratories. Ecol Evol. 2014; 4(18):3514–24. 10.1002/ece3.1155. PMC4224527 25478144

[20] Shade A. Diversity is the question not the answer. The ISME J. 2016; 11(1):1–6. 10.1038/ismej.2016.118. PMC5421358 27636395

[21] Massana R, Logares R. Eukaryotic versus prokaryotic marine picoplankton ecology. Environ Microbiol. 2012; 15(5):1254–61. 10.1111/1462-2920.12043. 23206217

[22] Grossmann L, Jensen M, Heider D, Jost S, Glücksman E, Hartikainen H, Mahamdallie S, Gardner M, Hoffmann D, Bass D, Boenigk J. Protistan community analysis: key findings of a large-scale molecular sampling. The ISME J. 2016; 10(9):2269–79. 10.1038/ismej.2016.10. PMC4989302 26859769

[23] Foster KR, Schluter J, Coyte KZ, Rakoff-Nahoum S. The evolution of the host microbiome as an ecosystem on a leash. Nature. 2017; 548(7665):43–51. 10.1038/nature23292. PMC5749636 28770836

[24] Boenigk J, Wodniok S, Bock C, Beisser D, Hempel C, Grossmann L, Lange A, Jensen M. Geographic distance and mountain ranges structure freshwater protist communities on a european scale. Metabarcoding and Metagenomics. 2018; 2:21519. 10.3897/mbmg.2.21519.

[25] Yi Z, Berney C, Hartikainen H, Mahamdallie S, Gardner M, Boenigk J, Cavalier-Smith T, Bass D. High throughput sequencing of microbial eukaryotes in lake baikal reveals ecologically differentiated communities and novel evolutionary radiations. FEMS Microbiol Ecol. 2017. 10.1093/femsec/fix073. 28575320

[26] Jani K, Dhotre D, Bandal J, Shouche Y, Suryavanshi M, Rale V, Sharma A. World’s largest mass bathing event influences the bacterial communities of godavaria holy river of india. Microb Ecol. 2018. 10.1007/s00248-018-1169-1. 29536131

[27] Guimarães PR, Pires MM, Jordano P, Bascompte J, Thompson JN. Indirect effects drive coevolution in mutualistic networks. Nature. 2017; 550(7677):511–14. 10.1038/nature24273. 29045396

[28] Oliverio AM, Power JF, Washburne A, Cary SC, Stott MB, Fierer N. The ecology and diversity of microbial eukaryotes in geothermal springs. The ISME J. 2018. 10.1038/s41396-018-0104-2. PMC6052046 29662145

[29] Rossum TV, Peabody MA, Uyaguari-Diaz MI, Cronin KI, Chan M, Slobodan JR, Nesbitt MJ, Suttle CA, Hsiao WWL, Tang PKC, Prystajecky NA, Brinkman FSL. Year-long metagenomic study of river microbiomes across land use and water quality. Front Microbiol. 2015; 6. 10.3389/fmicb.2015.01405. PMC4681185 26733955

[30] Zeglin LH. Stream microbial diversity in response to environmental changes: review and synthesis of existing research. Front Microbiol. 2015; 6. 10.3389/fmicb.2015.00454. PMC4435045 26042102

[31] Tanaka D, Takahashi T, Yamashiro Y, Tanaka H, Kimochi Y, Nishio M, Sakatoku A, Nakamura S. Seasonal variations in bacterioplankton community structures in two small rivers in the himi region of central japan and their relationships with environmental factors. World J Microbiol Biotechnol. 2017; 33(12). 10.1007/s11274-017-2377-4. 29134451

[32] Dudgeon D, Arthington AH, Gessner MO, Kawabata Z-I, Knowler DJ, Lévêque C, Naiman RJ, Prieur-Richard A-H, Soto D, Stiassny MLJ, Sullivan CA. Freshwater biodiversity: importance, threats, status and conservation challenges. Biol Rev. 2005; 81(02):163. 10.1017/s1464793105006950. 16336747

[33] Seto KC, Fragkias M, Gneralp B, Reilly MK. A meta-analysis of global urban land expansion. PLoS ONE. 2011; 6(8):23777. 10.1371/journal.pone.0023777. PMC3158103 21876770

[34] Waters CN, Zalasiewicz J, Summerhayes C, Barnosky AD, Poirier C, uszka AG, Cearreta A, Edgeworth M, Ellis EC, Ellis M, Jeandel C, Leinfelder R, McNeill JR, d Richter D, Steffen W, Syvitski J, Vidas D, Wagreich M, Williams M, Zhisheng A, Grinevald J, Odada E, Oreskes N, Wolfe AP. The anthropocene is functionally and stratigraphically distinct from the holocene. Science. 2016; 351(6269):2622–2622. 10.1126/science.aad2622. 26744408

[35] Isbell F, Gonzalez A, Loreau M, Cowles J, Díaz S, Hector A, Mace GM, Wardle DA, O’Connor MI, Duffy JE, Turnbull LA, Thompson PL, Larigauderie A. Linking the influence and dependence of people on biodiversity across scales. Nature. 2017; 546(7656):65–72. 10.1038/nature22899. PMC5460751 28569811

[36] Macher J-N, Leese F. Environmental DNA metabarcoding of rivers: Not all edna is everywhere and not all the time. 2017. bioRxiv. 10.1101/164046. http://arxiv.org/abs/http://www.biorxiv.org/content/early/2017/07/15/164046.full.pdf.

[37] Thompson LR, Sanders JG, McDonald D, Amir A, Ladau J, Locey KJ, Prill RJ, Tripathi A, Gibbons SM, Ackermann G, Navas-Molina JA, Janssen S, Kopylova E, Vázquez-Baeza Y, González A, Morton JT, Mirarab S, Xu ZZ, Jiang L, Haroon MF, Kanbar J, Zhu Q, Song SJ, Kosciolek T, Bokulich NA, Lefler J, Brislawn CJ, Humphrey G, Owens SM, Hampton-Marcell J, Berg-Lyons D, McKenzie V, Fierer N, Fuhrman JA, Clauset A, Stevens RL, Shade A, Pollard KS, Goodwin KD, Jansson JK, Gilbert JA, Knight R, Consortium TEMP. A communal catalogue reveals earth’s multiscale microbial diversity. Nature. 2017. 10.1038/nature24621. PMC6192678 29088705

[38] Power JF, Carere CR, Lee CK, Wakerley GL, Evans DW, Button M, White D, Climo MD, Hinze AM, Morgan XC, McDonald IR, Cary SC, Stott MB. Microbial biogeography of 1,000 geothermal springs in New Zealand. 2018. bioRxiv. 10.1101/247759. http://arxiv.org/abs/https://www.biorxiv.org/content/early/2018/01/15/247759.full.pdf. PMC6056493 30038374

