# Supplementary figures and images for: SEDE-GPS: socio-economic data enrichment based on GPS information
Source: BMC Bioinformatics. 2018 Nov 30;19(Suppl 15):440. doi: 10.1186/s12859-018-2419-4 (PMC6266930; doi:10.1186/s12859-018-2419-4)

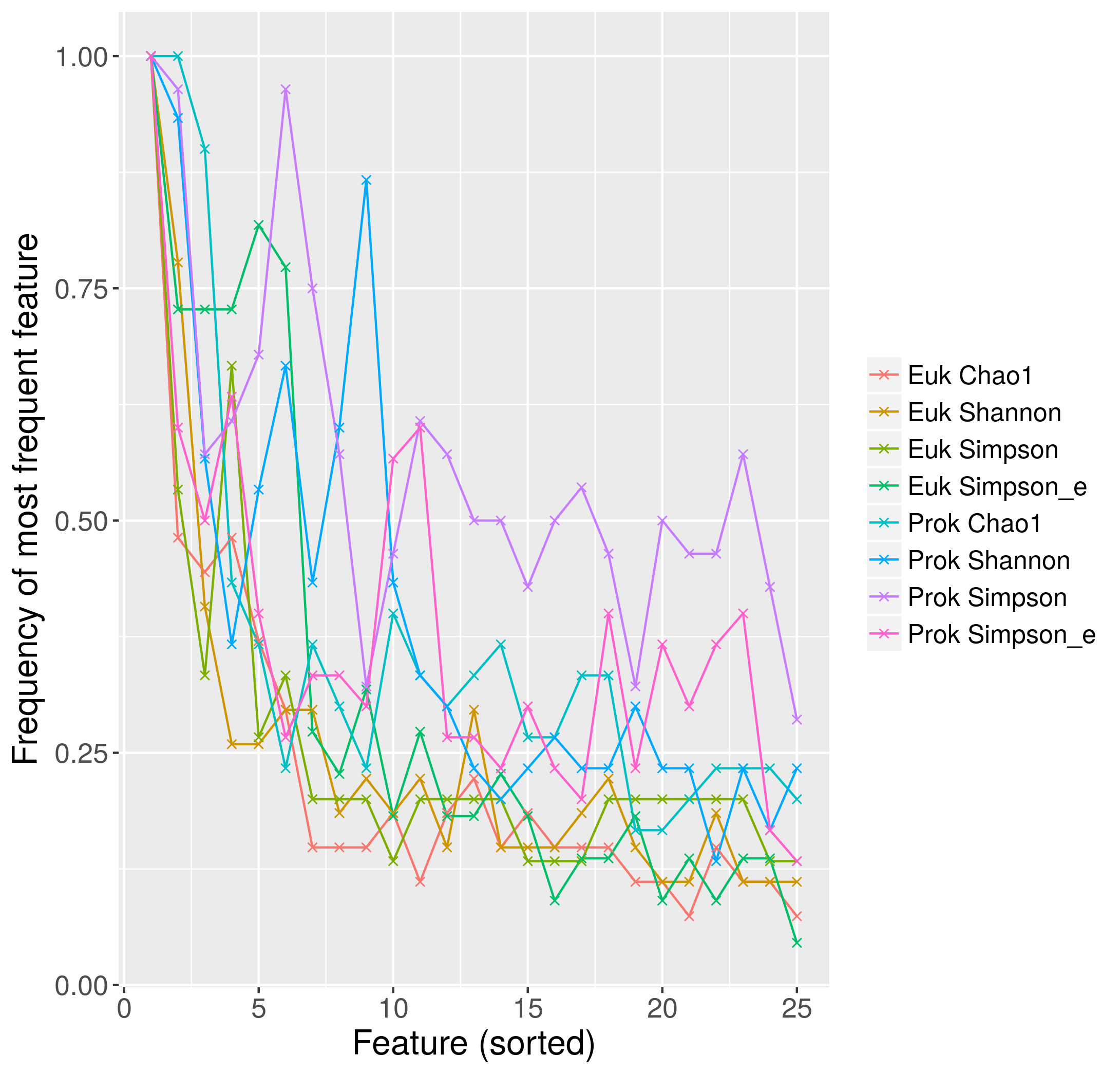

Supplement: Supplementary file 3 — This figure shows the relative frequency of the most frequent feature at a given position for all target variables. Frequencies were calculated from the feature lists sorted by the weights determined by EFS in the LOOCV iterations. This shows that feature lists get more random with increasing rank of the feature on a sorted feature list. (TIF 844 kb) [file 12859_2018_2419_MOESM3_ESM.tif]
